# Recombination analysis based on the complete genome of bocavirus

**DOI:** 10.1186/1743-422X-8-182

**Published:** 2011-04-20

**Authors:** Xingli Fu, Xiaochun Wang, Bin Ni, Hongxing Shen, Hua Wang, Xiaodan Zhang, Shengxia Chen, Shihe Shao, Wen Zhang

**Affiliations:** 1 School of Medical Technology, Jiangsu University, 301 Xuefu Road, Zhenjiang, Jiangsu 212013, PR China; 2 School of Medicine, Jiangsu University, 301 Xuefu Road, Zhenjiang, Jiangsu 212013, PR China

## Abstract

Bocavirus include bovine parvovirus, minute virus of canine, porcine bocavirus, gorilla bocavirus, and Human bocaviruses 1-4 (HBoVs). Although recent reports showed that recombination happened in bocavirus, no systematical study investigated the recombination of bocavirus. The present study performed the phylogenetic and recombination analysis of bocavirus over the complete genomes available in GenBank. Results confirmed that recombination existed among bocavirus, including the likely inter-genotype recombination between HBoV1 and HBoV4, and intra-genotype recombination among HBoV2 variants. Moreover, it is the first report revealing the recombination that occurred between minute viruses of canine.

## Introduction

Members of the genus *Bocavirus *are non-enveloped single-stranded DNA (ss-DNA) virus, which belong to the Parvoviridae family. The bocavirus genome is not segmented and contains a single molecule of linear, positive- or negative-sense DNA of 4,000-6,000 nucleotides in length [1]. Known members of bocavirus include bovine parvovirus (BPV), minute virus of canine (MVC), porcine bocavirus (PBoV), gorilla bocavirus (GBoV), and Human bocaviruses 1-4 (HBoV1-4).

The MVC genome shares about 43% identity with BPV over the genome level [2,3]. BPV was first identified in 1961 in samples from calves with diarrhea [4], while MVC was first isolated from canine fecal samples in 1970 [5]. HBoV was first described in pooled nasopharyngeal aspirates from children with respiratory infections in 2005, and was provisionally categorized into the genus bocavirus [1]. Subsequently, HBoV2, HBoV3, and HBoV4 were discovered, sharing a mean similarity of 80% with HBoV1, and all have been categorized into the genus bocavirus [6,7]. Recently, new bocavirus species were isolated from gorilla and swine, and most closely related to HBoV [8,9].

Although recently reports showed that recombination happened in bocavirus [8], no study has systematically investigated the recombination among bocavirus strains. In the present study, therefore, we analyzed the available complete bocavirus genome sequences in GenBank to elucidate the recombination among bocavirus strains.

## Methods

### Sequences

The study sequences comprised all the 121 available complete genome sequences of bocavirus from GenBank dated September 2010. Sequences were firstly screened to exclude patented and artificial mutants, and then aligned in the ClustalW program [10]. The alignment was manually adjusted for the correct reading frame. Sequences showing less than 1% divergence from each other were considered as the same. The remaining 54 BoV genomes included one GBoV, one BPV, two PBoVs, three MVCs, and 47 HBoVs.

### Phylogenetic analysis

Before phylogenetic analysis, multiple-alignment was performed in the ClustalW program. Phylogenetic trees were constructed using the neighbor-joining method and evaluated using the interior branch test method with Mega 4 software [11]. Percent bootstrap support was indicated at each node. GenBank accession no. was indicated at each branch.

### Recombination Detection

The remaining 54 bocavirus genomes were re-aligned in the ClustalW program. Detection of potential recombinant sequences, identification of potential parental sequences, and localization of possible recombination break points were determined using the Recombination Detection Program (RDP)[12], GENECONV [13], BOOTSCAN [14], MaxChi [15], CHIMAERA [16], and SISCAN [17] methods embedded in RDP3 [18]. A Multiple-comparison-corrected P-value cutoff of 0.05 was used throughout.

## Results and Discussion

**Figure 1:**
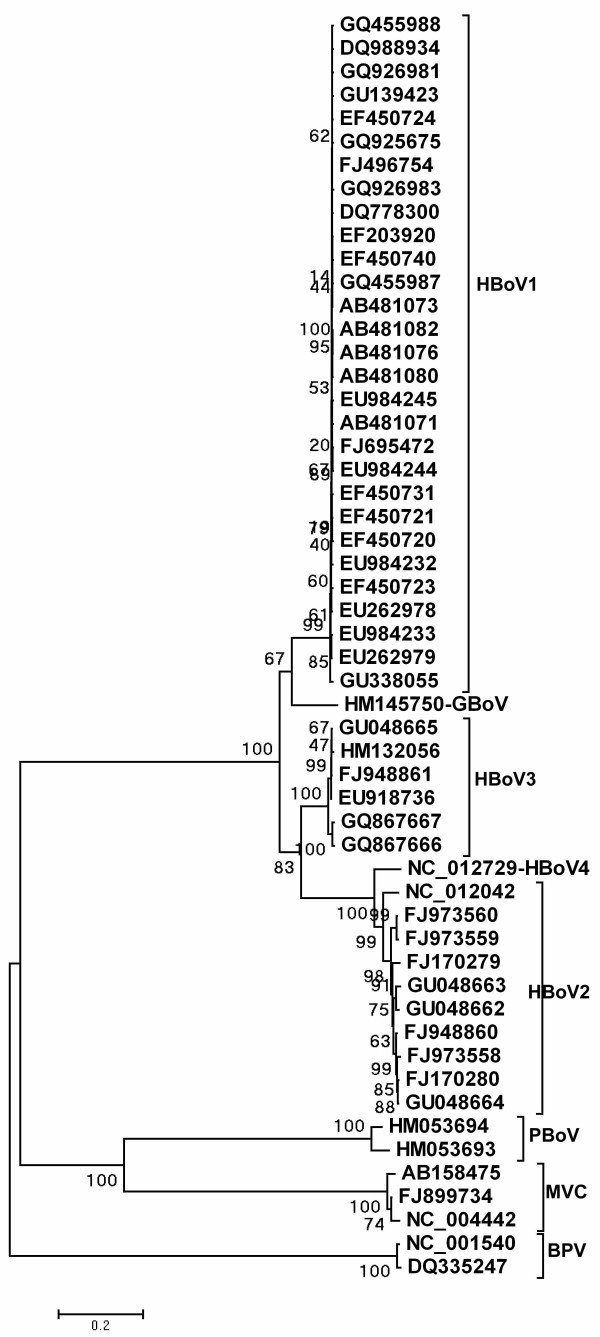
**Phylogenetic tree for the 54 complete bocavirus genomes**. Phylogenetic analysis were performed using the neighbor-joining method and evaluated using the interior branch test method with Mega 4 software. Values for various branches are percentages of the tree obtained from 1000 resamplings of the data. Percent bootstrap supports that higher than 50 are indicated at nodes.

Based on the 54 complete genomes, a phylogenetic tree was constructed (Figure 1). The genotypes of these bocavirus showed in the phylogenetic tree were consistent with the genotype information from the original sources. From the phylogenetic tree, we can see that HBoV2 and HBoV4 closely related and formed into one cluster; HBoV1 and HBoV3 clustered separately, and GBoV showed more related to HBoV1 than to the other HBoVs. Six potentially significant recombination events were found with a high degree of confidence (p value ≤ 4.4 × 10^-3^) judged by the above-mentioned six recombination detection methods.

**Figure 2:**
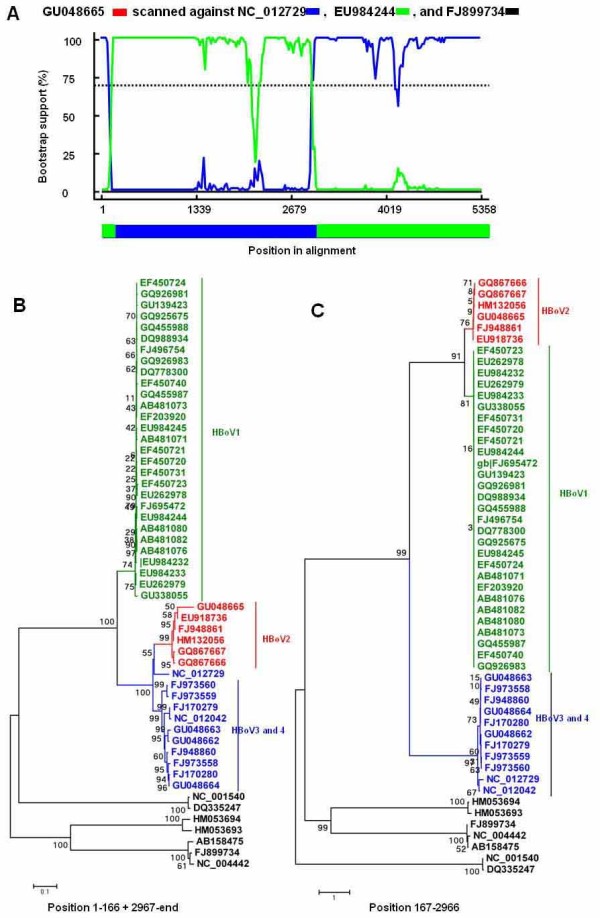
**Identification of recombination between HBoV1 and HBoV4, which led to the recombinant cluster of HBoV2**. (A) BOOTSCAN evidence for the recombination origin on the basis of pairwise distance, modeled with a window size 200, step size 20, and 100 Bootstrap replicates; (B) Neighbor joining tree (2,000 replicates, Kimura 2-parameter distance) constructed using the non-recombinant region (Position 1-166 + 2967-end); (C) Neighbor joining tree (2,000 replicates, Kimura 2-parameter distance) constructed using the recombinant region (Position 167-2966).

Figure 2 indicated the inter-genotype recombination events that occurred between HBoV1 and HBoV4, which lead to the recombinant cluster HBoV2. The BOOTSCAN plot of this recombination event was showed in Figure 2A, which used the lineages represented by NC_012729 and EU984244 as the parental strains, leading to the daughter lineage represented by GU048665. To confirm this recombination event, the relevant strains were analyzed by neighbor joining trees using MEGA4. Figure 2B and 2C were two trees constructed on the non-recombinant region (position: 1-166 + 2967-end) and the recombinant region (position:167-2966), respectively. In Figure 2B, all HBoV2 strains clustered closely with HBoV3 and HBoV4, while Figure 2C showed discordant phylogenetic relationships compared with Figure 2B, where HBoV2 strains clustered with HBoV1. The phylogenetic analysis confirmed the recombination event that occurred between HBoV1 and HBoV4, leading to the recombinant HBoV2.

**Figure 3:**
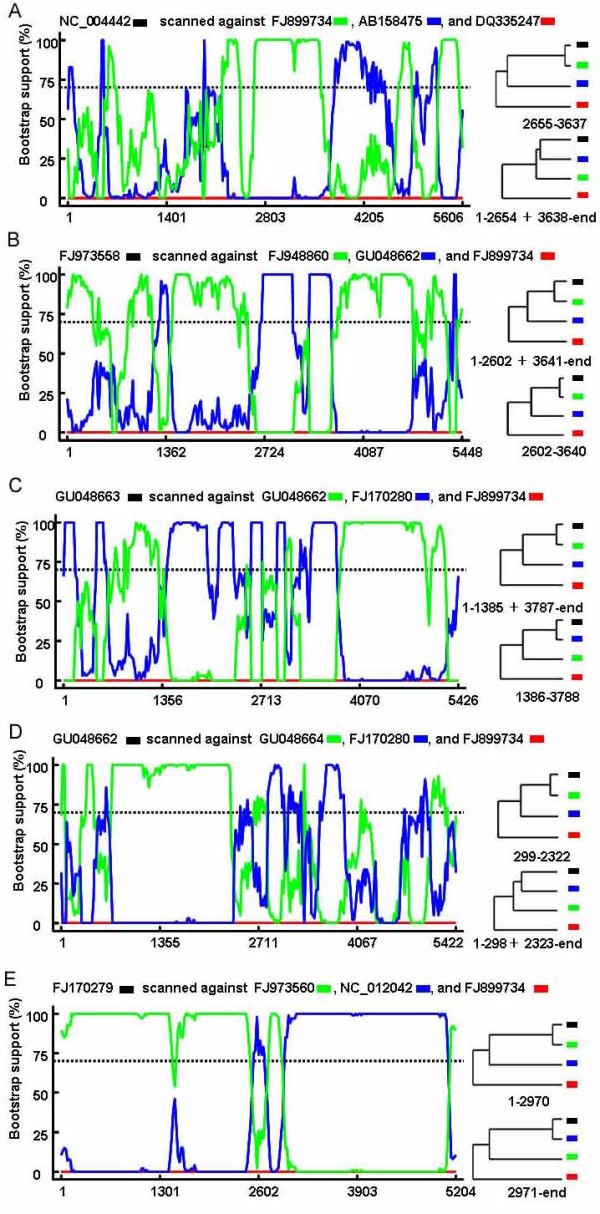
**Identification of the other 5 recombination events**. The left part of each panel was BOOTSCAN evidence for the recombination origin on the basis of pairwise distance, modeled with a window size 200, step size 20, and 100 Bootstrap replicates; The right part of each panel were phylogenetic trees constructed based on recombination regions and non-recombination regions using Mega 4 software.

Figure 3A-E indicated the other 5 recombinants identified in the present study. The BOOTSCAN plot of each recombination event was showed on the left side, while phylogenetic tree based on recombination regions and non-recombination regions of parental strains in each recombination event were indicated on the right side. Figure 3A showed the recombinant occurred between two MVC strains, FJ899734[19] and AB158475[20], leading to the recombinant NC_004442[3]. These recombination events provide the first evidence that recombination could happened among MVC strains. Figure 3B showed a recombination event happened intra-genotypely between two HBoV2 strains, FJ948860 and GU048662, leading to the recombinant FJ973558, another HBoV2 strain. FJ948860 and GU048662 were isolated from Australia in 2001 [7] and Thailand in 2007 according to the isolate information in GenBank, respectively, while FJ973558 was isolated from Tunisia in 2006 [8]. Figure 3C and 3D revealed two Bangpoo recombinants, GU048662 and GU048663, respectively. However, according to the isolate information in GenBank, GU048662, GU048663, and one of their parental isolates were isolated in the same lab in Thailand, therefore, it should be cared whether these recombination events non-naturally occurred by sequencing error and/or contamination in the lab. Figure 3E revealed recombination between the lineage represented by Nigeria HBoV2 isolate FJ973560 and Pakistan HBoV2 isolate lineage NC_012042, which led to a recombinant Pakistan isolate FJ170279. However, these three isolates were determined in the same lab [6,8], therefore, whether this recombination event non-naturally occurred by sequencing error and/or contamination should be confirmed by further study.

For RNA viruses, recombination has been shown to be an important feature of their evolution [21-23], and single-stranded DNA parvoviruses have been shown to have a mutation rate approaching that of RNA viruses [24]. The HBoV sequences were previously considered to show very low protein and nucleotide sequence diversity [25]. Recently, however, other 3 different genotypes (HBoV2-4) were discovered, which reveals that the HBoVs own the property of high sequence diversity. The present study provide the evidence that recombination was observed through complete bocavirus genome analyses, including the likely inter-genotype recombination between HBoV1 and HBoV4, and intra-genotype recombination between HBoV2 variants. As a matter of fact, a recent study indicated HBoVs coinfection was detected [8], which will increase the chance of recombination between baocavirus strains.

## Conclusion

Taken together, this study confirmed that recombination existed among bocavirus, including the likely inter-genotype recombination between HBoV1 and HBoV4, and intra-genotype recombination among HBoV2 variants. It is the first report revealing the recombination that occurred between minute virus strains of canine.

## Competing interests

The authors declare that they have no competing interests.

## Authors' contributions

WZ and XF conceived the study. WZ and XF wrote the paper. All authors performed recombination analysis and critically reviewed and approved the final manuscript.

## References

[B1] Allander T Tammi MT Eriksson M Bjerkner A Tiveljung-Lindell A Andersson B Cloning of a human parvovirus by molecular screening of respiratory tract samples Proc Natl Acad Sci USA 2005 102 12891 12896 10.1073/pnas.0504666102 16118271 PMC1200281

[B2] Sun Y Chen AY Cheng F Guan W Johnson FB Qiu J Molecular characterization of infectious clones of the minute virus of canines reveals unique features of bocaviruses J Virol 2009 83 3956 3967 10.1128/JVI.02569-08 19211770 PMC2663281

[B3] Schwartz D Green B Carmichael LE Parrish CR The canine minute virus (minute virus of canines) is a distinct parvovirus that is most similar to bovine parvovirus Virology 2002 302 219 223 10.1006/viro.2002.1674 12441065

[B4] Abinanti FR Warfield MS Recovery of a hemadsorbing virus (HADEN) from the gastrointestinal tract of calves Virology 1961 14 288 289 10.1016/0042-6822(61)90206-9 13681037

[B5] Binn LN Lazar EC Eddy GA Kajima M Recovery and Characterization of a Minute Virus of Canines Infect Immun 1970 1 503 508 16557766 10.1128/iai.1.5.503-508.1970 PMC415932

[B6] Kapoor A Slikas E Simmonds P Chieochansin T Naeem A Shaukat S Alam MM Sharif S Angez M Zaidi S Delwart E A newly identified bocavirus species in human stool J Infect Dis 2009 199 196 200 10.1086/595831 19072716 PMC2678954

[B7] Arthur JL Higgins GD Davidson GP Givney RC Ratcliff RM A novel bocavirus associated with acute gastroenteritis in Australian children PLoS Pathog 2009 5 e1000391 10.1371/journal.ppat.1000391 19381259 PMC2663820

[B8] Kapoor A Simmonds P Slikas E Li L Bodhidatta L Sethabutr O Triki H Bahri O Oderinde BS Baba MM Bukbuk DN Besser J Bartkus J Delwart E Human bocaviruses are highly diverse, dispersed, recombination prone, and prevalent in enteric infections J Infect Dis 2010 201 1633 1643 10.1086/652416 20415538 PMC2902747

[B9] Cheng WX Li JS Huang CP Yao DP Liu N Cui SX Jin Y Duan ZJ Identification and nearly full-length genome characterization of novel porcine bocaviruses PLoS One 2010 5 e13583 10.1371/journal.pone.0013583 21049037 PMC2963602

[B10] Thompson JD Higgins DG Gibson TJ CLUSTAL W: improving the sensitivity of progressive multiple sequence alignment through sequence weighting, position-specific gap penalties and weight matrix choice Nucleic Acids Res 1994 22 4673 4680 10.1093/nar/22.22.4673 7984417 PMC308517

[B11] Tamura K Dudley J Nei M Kumar S MEGA4: Molecular evolutionary genetics analysis (MEGA) software version 4.0 Mol Biol Evol 2007 24 1596 1599 10.1093/molbev/msm092 17488738

[B12] Martin D Rybicki E RDP: detection of recombination amongst aligned sequences Bioinformatics 2000 16 562 563 10.1093/bioinformatics/16.6.562 10980155

[B13] Padidam M Sawyer S Fauquet CM Possible emergence of new geminiviruses by frequent recombination Virology 1999 265 218 225 10.1006/viro.1999.0056 10600594

[B14] Martin DP Posada D Crandall KA Williamson C A modified bootscan algorithm for automated identification of recombinant sequences and recombination breakpoints AIDS Res Hum Retrovir 2005 21 98 102 10.1089/aid.2005.21.98 15665649

[B15] Smith JM Analyzing the mosaic structure of genes J Mol Evol 1992 34 126 9 1556748 10.1007/BF00182389

[B16] Posada D Crandall KA Evaluation of methods for detecting recombination from DNA sequences: computer simulations Proc Natl Acad Sci USA 2001 98 13757 13762 10.1073/pnas.241370698 11717435 PMC61114

[B17] Gibbs MJ Armstrong JS Gibbs AJ Sister-scanning: a Monte Carlo procedure for assessing signals in recombinant sequences Bioinformatics 2000 16 573 582 10.1093/bioinformatics/16.7.573 11038328

[B18] Martin DP Williamson C Posada D RDP2: recombination detection and analysis from sequence alignments Bioinformatics 2005 21 260 262 15377507 10.1093/bioinformatics/bth490

[B19] Shan TL Cui L Dai XQ Guo W Shang XG Yu Y Zhang W Kang YJ Shen Q Yang ZB Zhu JG Hua XG Sequence analysis of an isolate of minute virus of canines in China reveals the closed association with bocavirus Mol Biol Rep 2010 37 2817 2820 10.1007/s11033-009-9831-9 19760094

[B20] Ohshima T Kishi M Mochizuki M Sequence analysis of an Asian isolate of minute virus of canines (canine parvovirus type 1) Virus Genes 2004 29 291 296 10.1007/s11262-004-7430-3 15550767

[B21] Malim MH Emerman M HIV-1 sequence variation: drift, shift, and attenuation Cell 2001 104 469 474 10.1016/S0092-8674(01)00234-3 11239404

[B22] Simmonds P Recombination and selection in the evolution of picornaviruses and other Mammalian positive-stranded RNA viruses J Virol 2006 80 11124 1140 10.1128/JVI.01076-06 16956935 PMC1642140

[B23] Liu W Zhai J Liu J Xie Y Identification of recombination between subgenotypes IA and IB of hepatitis A virus Virus Genes 2010 40 222 224 10.1007/s11262-009-0431-5 20012679

[B24] Duffy S Shackelton LA Holmes EC Rates of evolutionary change in viruses: patterns and determinants Nat Rev Genet 2008 9 267 276 18319742 10.1038/nrg2323

[B25] Kesebir D Vazquez M Weibel C Shapiro ED Ferguson D Landry ML Kahn JS Human bocavirus infection in young children in the United States: molecular epidemiological profile and clinical characteristics of a newly emerging respiratory virus. J Infect Dis 2006 194 1276 1282 10.1086/508213 PMC7204143 17041854

